# Size-dependent sex allocation and reproductive investment in a gynodioecious shrub

**DOI:** 10.1093/aobpla/plw089

**Published:** 2016-12-30

**Authors:** Akari Shibata, Gaku Kudo

**Affiliations:** a Graduate School of Environmental Earth Science, Hokkaido University, Sapporo, 060-0810, Japan

**Keywords:** Cost of reproduction, flower production, fruit set, gynodioecy, pollen limitation, resource allocation, sexual dimorphism, size-dependency

## Abstract

In sexually dimorphic plants, resource allocation to reproduction often differs between sex morphs. In gynodioecious species, *i.e.* coexisting hermaphrodite and female plants within a population, females often produce more fruits than hermaphrodites. Since fruit production is costlier than flower production, hermaphrodites and females may regulate flower and fruit production differently in response to resource availability. To clarify the gender-specific strategies of reproductive allocation, we assessed sexual dimorphism in reproductive traits, size-dependent resource allocation, morphological traits, and photosynthetic capacity in a natural population of a gynodioecious shrub, *Daphne jezoensis*. Hermaphrodites had larger flowers and increased flower number with plant size at a rate greater than females, but showed consistently smaller fruit production. Although females did not increase flower production as much as hermaphrodites did as their size increased, they produced 3.7 times more fruits than did hermaphrodites. Despite a large sexual difference in fruiting ability based on hand-pollination, total resource investment in reproduction (the sum of flower and fruit mass) was similar between sex morphs across plant sizes, and there was a little sexual difference in the cost of reproduction, *i.e.* the negative effect of current reproduction on future reproductive effort, in the natural population. In addition, there were no sexual differences in the resource allocation to vegetative organs (leaf and root mass) and photosynthetic capacity (light response photosynthetic rates). Under natural conditions, pollen limitation strongly restricted the fruit production of females, resulting in similar cost of reproduction between hermaphrodites and females.

## Teaser

Gynodioecy is a mating system where both hermaphrodite (pollen and seed producer) and female plants (seed producer) coexist within a population. We compared size-dependent sex allocation between hermaphrodites and females in a gynodioecious shrub, Daphne jezoensis. Hermaphrodites produced more flowers with size increment than females, while females produced 3.7 times more fruits than hermaphrodites. Despite the sexual difference in sex allocation, total reproductive investment did not differ between the sex morphs because of low pollination success. Severe pollen limitation under natural conditions resulted in the balanced resource investment in reproduction between the sex morphs in this species.

## Introduction

Gynodioecy is a mating system where both hermaphrodites and male-sterile plants (females) coexist within a population. Approximately 7 % of angiosperm species exhibit this mating system (Richards 1997). Gynodioecy is considered to be an intermediate stage in the evolution of dioecy (males and females only) from hermaphroditism, and understanding this transition is crucial to clarifying the evolutionary pathways that have led to separate sexes in plants (Charlesworth and Charlesworth 1978; Spigler and Ashman 2012; Dufay *et al.* 2014). In gynodioecious species, hermaphrodites can gain fitness through pollen and seed production, while females gain fitness only through seed production. Theoretically, females must compensate this reproductive disadvantage, *i.e.* lack of fitness as a pollen donor, in order to invade and remain in hermaphroditic populations (Lewis 1941; Charlesworth and Charlesworth 1978). In actuality, females often produce more, or higher quality, seeds than hermaphrodites in many gynodioecious species (Shykoff *et al.* 2003; Dufay and Billard 2012). To understand the maintenance mechanism of gynodioecy and its evolutionary status, comparisons of female fecundity and resource investment in reproduction between sex morphs are crucial (Delph 1990; Delph and Wolf 2005; Spigler and Ashman 2012).

Hermaphrodites and females may have a different resource allocation strategy for reproduction (Ashman 2006). Females simply increase resource investment in fruit production as plant size (or resource availability) increases, while hermaphrodites may regulate resource allocation between flower (pollen) and fruit (seed) production depending on resource availability (Delph 1990; Ashman 1999). Size- or resource-dependent allocation to male and female functions has been observed in hermaphrodites of several gynodioecious and sub-dioecious species, where the male function of hermaphrodites tends to be enhanced in small-sized plants or under low-resource conditions, indicating plastic sexual function of hermaphrodites (Delph 1990; Delph and Wolf 2005). If the plasticity of seed fertility is costly and environmentally dependent, however, the relative male function of hermaphrodites may vary among populations, independent of plant size, reflecting site-specific resource conditions and genetic variation among populations (Ashman 2006). Therefore, comparisons of size-dependent reproductive performance between hermaphrodites and females are important for the clarification of gender-specific resource allocation strategies.

Large resource investment in the current reproduction cycle may cause negative effects on future reproduction, growth, or survival, *i.e.* the cost of reproduction (Obeso 2002). Sexual differences in the cost of reproduction have been reported in several dioecious species (Lloyd and Webb 1977; Delph 1999; Obeso 2002; Álvarez-Cansino *et al.* 2010), in which females usually invest larger resources in reproduction than males because of the cost involved in fruit production. Similarly, in gynodioecious species, resource investment in reproduction may be greater in females than in hermaphrodites, when females produce more fruits (Kohn 1989; Delph 1990; Vaughton and Ramsey 2011; Toivonen and Mutikainen 2012). Under natural conditions, however, the fruit production of animal pollinated plants is often restricted by pollen limitation (Knight *et al.* 2005). Less fruit production under the frequent pollen limitation may reduce the sexual difference in the cost of reproduction. Furthermore, whether the degree of pollen limitation varies between sex morphs is crucial to determine the sexual differences in reproductive investment in gynodioecious species (Asikainen and Mutikainen 2005; Wang *et al.* 2014). Therefore, comparisons of the cost of reproduction between sex morphs under natural conditions are important in clarifying how hermaphrodites and females are maintained in the population.

Sexual dimorphism in reproductive traits, such as flower size, flower number, and fruiting ability, is widely reported in both dioecious and gynodioecious plants (Delph 1999; Eckhart 1999; Shykoff *et al.* 2003). In contrast, the trend in vegetative traits, such as leaf size, leaf number, and assimilation ability, is different: dioecious species commonly exhibit dimorphism in vegetative traits between males and females, while gynodioecious species tend to have modest differences between hermaphrodites and females (Schultz 2003, 2009; Ashman 2005). This may indicate that dimorphism in vegetative traits evolves after the evolution of reproductive traits responding to sexual selection (Geber 1999). However, since there are only a few studies on dimorphism in vegetative traits of gynodioecious species (Ashman 2005; Schultz 2009), additional information is needed to clarify the general features of sexual dimorphism in gynodioecy, especially at the whole plant level, including belowground parts.

In this study, we aimed to assess sexual dimorphism in reproductive performance, size-dependent resource allocation, and morphological and physiological traits in a gynodioecious shrub, *Daphne jezoensis* (Thymelaeaceae). Specifically, we address the following questions: (1) How do hermaphrodites and females vary allocation to flowers and fruits as plant size increases? (2) Is there any difference in total resource investment in reproduction and the cost of reproduction between sex morphs, and to what extent is fruit production restricted by pollen limitation under natural conditions? and (3) Is there any sexual dimorphism in vegetative traits, *i.e.* morphology, resource allocation to vegetative organs, and photosynthetic capacity?

## Methods

### Study species

*D. jezoensis* (synonym, *D. kamtschatica* var. *jezoensis*) is a summer deciduous shrub, distributed throughout the deciduous forests of northern Japan. Flowering occurs in early spring, soon after snowmelt. Flowers persist through to late May, and the fruits mature in mid-summer (July). Each fruit contains a single seed. Leaf senescence occurs as the forest canopy closes, usually in mid-June, and plants remain dormant for 2 months. New leaves flush in late August. Floral buds develop during autumn and are retained throughout the winter. Tubular-shaped flowers produce small amount of nectar, and are visited by moths, skippers and bumblebees at low frequency. Although small pollen beetles and thysanopteran insects are observed on flowers, they appear to be pollen consumers rather than pollinators (personal observation). *D. jezoensis* is known as a gynodioecious plant, with two distinct floral morphs (Kikuzawa 1989). Hermaphrodite flowers have large anthers, and four of eight anthers protrude from the floral tube. Female flowers have highly reduced anthers and do not contain pollen. Generally, hermaphrodites have lower fruit production than females. Approximately 40 % of hermaphrodites did not set any fruit during a 6-year census in one population (Sinclair *et al.* 2016). Thus, hermaphrodites appear to gain more fitness as pollen donors than as seed producers (Sinclair *et al.* 2016).

### Study site

This study was conducted at the Tomakomai experimental forest of Hokkaido University (TOEF; 42°40’ N, 141°36’ E; 70–80 m elevation) in Hokkaido, northern Japan. The mean monthly temperature ranges from 4.1 °C (January) to 20.3 °C (August), and annual precipitation is 1228 mm. Snow usually covers the ground from mid-December to late March, and the average snow depth is about 50 cm. A 50 m × 50 m permanent plot was established in a secondary deciduous forest in the spring of 2015, and all plants with floral buds (603 plants) were tagged and mapped. The frequency of females was 40.8 % in this population. The frequency of females ranged from 38 to 52 % in another 6 populations studied by Sinclair *et al.* 2016.

### Plant size, flower and fruit production in the natural population

The flower and fruit production of each tagged plant was recorded in 2015. Flowers were counted in the middle of the flowering period (late April to early May), and developing fruits were counted in mid-June before dispersal. One matured fruit was arbitrarily harvested from each of 34 individuals (31 females and 3 hermaphrodites) in July, oven-dried at 60 °C for at least 3 days, and individual fruit mass was weighed using a digital scale. To quantify the size of individual plants, the number of stems, stem diameter at the soil surface level, and stem length (from the ground to the tip of plant) were measured for 363 individuals (149 females and 214 hermaphrodites) in November 2015 using a calliper and flexible measuring tape.

Total reproductive investment per plant, *i.e.* the sum of flower and fruit mass, was calculated based on the flower and fruit number recorded, and the mean dry mass of individual flowers and fruits obtained at TOEF.

### Effect of current reproduction on future reproductive effort

The cost of reproduction under natural conditions, defined as a negative effect of current-year investment in reproduction on the next-year reproductive effort was examined for tagged plants. Current reproductive investment was estimated as described in the previous section. To quantify the next-year reproductive effort, floral buds that will open in the following spring were counted in October 2015.

### Fruit production under hand-pollination

For the evaluation of seed fertility (potential seed production) and pollen limitation of hermaphrodites and females under natural conditions, we conducted a hand-pollination experiment in the spring of 2016. We randomly chose 46 hermaphrodites and 22 females with floral buds in the plot, and covered one inflorescence on each plant with a fine-meshed bag to prevent pollination. Soon after opening before pollen dispersal in hermaphrodite flowers, pollen from multiple (5–10) hermaphrodite donors, harvested from at least a 10 m distance from the recipient plants, was artificially deposited on the stigmas of the covered inflorescences of 22 hermaphrodites and 22 females as a cross-pollination treatment. As a self-pollination treatment, we deposited own pollen on the stigmas of another 24 hermaphrodites. After recording the number of flowers treated, we re-covered the inflorescences with a fine-meshed bag to avoid uncontrolled pollination and herbivore damage. When fruits matured in mid-July, the number of fruits containing a developed seed was recorded. We calculated the fruit-set rate as a proportion of flowers setting fruits. Using the fruit-set rate under both natural pollination (NP) and cross-pollination (CP), we estimated the pollen limitation (PL) as PL = (CP–NP)/CP.

### Resource allocation and morphological traits

In order to compare sexual differences in resource allocation between plants, 21 plants of a similar size of each sex morph were harvested, including belowground parts, during the flowering period in 2015. We selected flowering plants with a single stem outside of the permanent plot. Harvested plants were separated into four parts (flower, leaf, stem, root) and oven-dried at 60 °C for at least 3 days. Before drying, the numbers of flowers and leaves were counted, the leaves were scanned for area measurement using a scanner, and flower width and tube length were measured using a digital calliper. Since the measurement of fruit production was impossible at the flowering stage, we estimated the expected number of fruits per plant (*Fr_n_*) from the number of flowers (*Fl_n_*) using the following formulas that were obtained in the TOEF population in 2015 (see RESULTS);

$Fr_{n}=\;\text{exp}\;\;\left(–2.57+1.03\times ln\;Fl_{n}\right)$ for female, and

$Fr_{n}=\;\text{exp}\;\;\left(–2.57+1.03\times ln\;Fl_{n}–1.78\right)$ for hermaphrodite.

The total fruit mass per plant was calculated by multiplying the estimated fruit number by the mean fruit weight obtained in the population (72.2 mg dry weight).

### Photosynthetic capacity

To assess the sexual difference in photosynthetic capacity, we measured the light responses of photosynthetic rates per unit leaf area in mid-May 2016 (end of flowering period) using a LI-6400 portable photosynthesis system (Li-Cor, Lincoln, NB, USA). This species has 2 types of leaves; autumn leaves that flush in late summer and retained until next late spring (hereafter overwintered leaves) and spring leaves that flush in spring and retained until early summer. Thus, 2 leaf cohorts (overwintered and spring leaves) coexist in the reproductive season, and we measured photosynthetic capacity of both cohorts. For three plants randomly selected in each sex morph, 10 light conditions (2000, 1500, 1000, 500, 300, 150, 100, 50, 10, and 0 µmol m^−^^2^ s^−^^1^) of PAR were tested using a red-blue LED light source at 15 °C under 380 µl m^−^^2^ CO_2_ conditions. The net photosynthetic rate per area *P_area_* can be described as a nonrectangular hyperbola of photon irradiance (*I*, µmol m^−^^2^ s^−^^1^) as follows:

$$P_{area}=\frac{\alpha l+P_{max}-\sqrt{{\left(\alpha l+P_{max}\right)}^{2}-4\alpha l\theta P_{max}}}{2\theta }-R_{d},$$

where *P_max_* is a light-saturated photosynthetic rate per unit area (µmol m^−^^2^ s^−^^1^), *α* is an initial slope (µmol m^−^^2^ s^−^^1^), *θ* is a degree of curvature, and *R_d_* is a dark respiration rate (µmol m^−^^2^ s^−^^1^). The data obtained for each sex morph was fitted to this equation by non-linear least-squares estimates of the parameters. Photosynthetic capacities of 2 of 6 leaves were unusually low, and we excluded them from the comparison.

### Statistical analyses

Sexual differences in plant size, reproductive traits and vegetative traits were assessed using generalised linear models (GLMs) in which sex morph was an explanatory variable. In the analysis of numerical traits (flower, fruit and leaf number), we used a GLM, postulating a negative binomial error distribution with log-link function to reduce overdispersion (O’Hara and Kotze 2010). In the analysis of continuous variables (stem volume, flower, leaf and root mass, flower morphology and leaf size), we used a GLM, postulating a gamma error distribution with log-link function. In the analysis of fruit-set rate under natural conditions, we used a GLM, postulating a binomial error distribution.

The relationship between flower number and fruit number was analysed using a GLM in which fruit number was a response variable, and flower number and sex morph were explanatory variables. Size-dependent flower and fruit production were analysed using a GLM in which flower and fruit number were each response variables, and stem volume and sex morph were explanatory variables. In all models, interactions between sex and other explanatory variables were considered.

In the GLM for the cost of reproduction, flower number for next spring (the number of floral buds in October 2015) was a response variable, and current reproductive investment (sum of flower and fruit mass), stem volume and sex morph were explanatory variables. Flower number in the current year (spring of 2015) was set as an offset term after logarithmic transformation to assess the changes in relative flower production in each plant. Interactions between sex and other explanatory variables were included in the model.

In the analysis of the hand-pollination experiment, we used a GLM, postulating a binomial error distribution in which fruit-set rate was a response variable and pollination treatment (natural- vs. cross-pollination or cross- vs. self-pollination) was an explanatory variable for each sex morph.

For the evaluation of size-dependent resource allocation, we analysed the following relationships using GLMs: (1) flower mass vs. vegetative mass (sum of leaf, stem, and root mass), (2) total reproductive mass (sum of flower and estimated fruit mass) vs. vegetative mass, (3) assimilative mass (leaf mass) vs. woody mass (sum of stem and root mass), and (4) root mass vs. shoot mass (sum of leaf and stem mass). In these models, interactions between sex morph and size index (vegetative mass, woody mass, or shoot mass) were considered. The fruit mass of individual plants was estimated based on the fruit number–flower number relationships described above. R version 3.2.3 was used for all statistical analyses (R Core Team 2015). For each GLM, a best-fit model was selected based on the Akaike’s information criterion (AIC).

## Results

### Plant size, flower, and fruit production in the natural population

**Figure 1:**
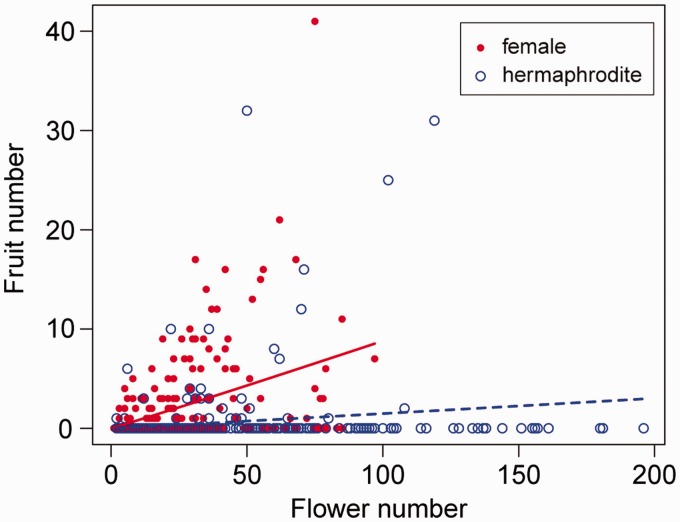
Relationships between flower number and fruit number in hermaphrodite (broken line) and female (solid line) plants under natural pollination. The line indicates the predicted relationship in each sex morph by GLM.

Stem volume, as a substitute for plant size, did not differ between the sex morphs in this population, but all reproductive traits showed significant sexual differences (Table 1). The mean flower number of hermaphrodites was 1.6 times greater than that of females. In contrast, females produced 3.7 times more fruits than did hermaphrodites. Females also had a 5.6 times higher fruit-set rate, although the fruit-set rate under natural conditions was low (less than 10 %) for both sex morphs. Fruit number significantly increased with flower number (*z* = 7.33, *P *< 0.001; Fig. 1), but this correlation was weaker in hermaphrodites (*z* = –6.88, *P *< 0.001).

**Table 1:** Comparisons of plant size and reproductive traits for hermaphrodite (H) and female plants (F) in the natural population. Mean ± SE (sample size) is indicated with GLM results.

	*F*	H	*t*, *z* value*	*P* value
**Plant size**				
Stem volume (cm^3^)	23.3 ± 2.0 (149)	24.3 ± 1.6 (214)	0.41	0.68
**Reproductive traits**				
Flower No.	24.4 ± 1.3 (246)	39.3 ± 1.9 (357)	6.80	**<0.001**
Fruit No.	2.2 ± 0.3 (246)	0.6 ± 0.2 (357)	–5.05	**<0.001**
Fruit-set rate (%)	8.4 ± 0.9 (246)	1.5 ± 0.4 (357)	–22.51	**<0.001**

* *t* value for plant size, *z* value for reproductive traits.

**Figure 2:**
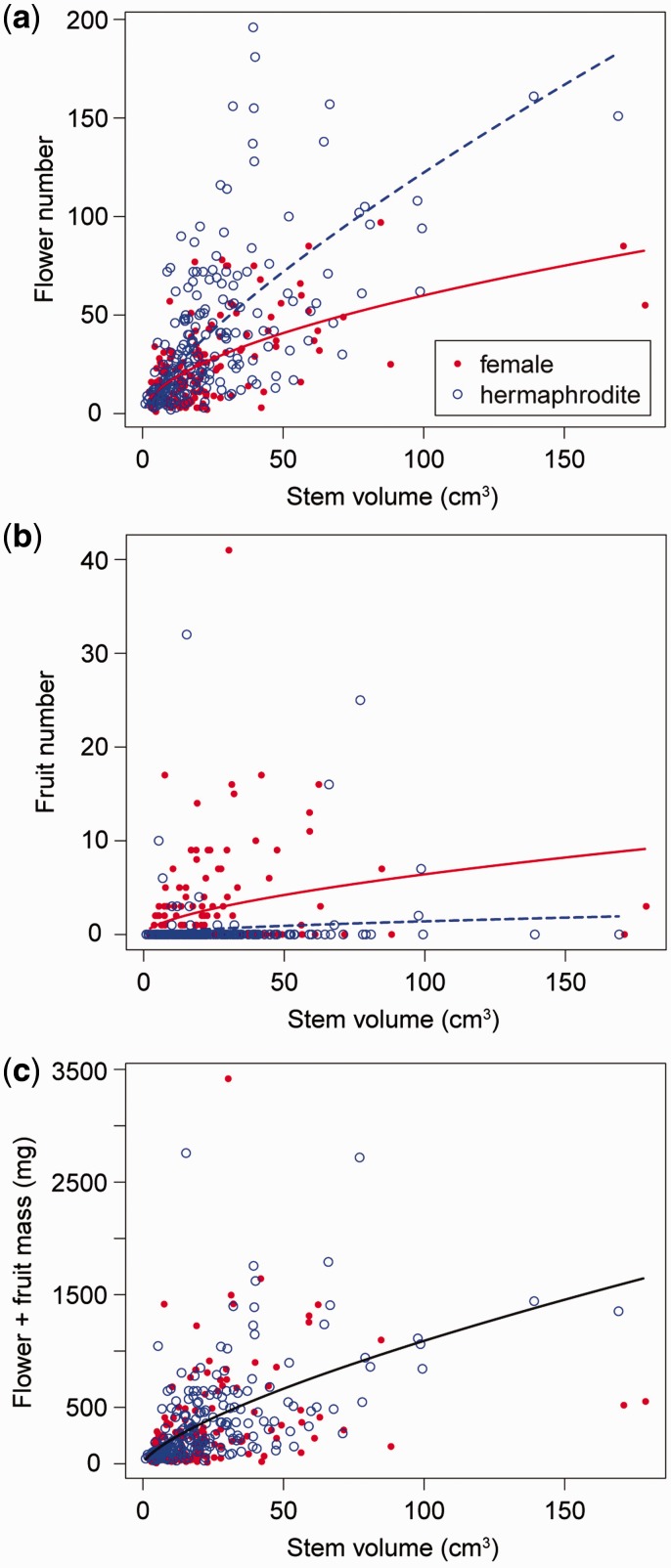
Relationships between stem volume and flower number (A), between stem volume and fruit number (B), and between stem volume and total reproductive investment (sum of flower and fruit mass) for hermaphrodite (broken line) and female (solid line) plants (C). Lines indicate the predicted relationship in each sex morph by GLMs independently (A), and by GLM including all individuals (B). Significant sexual difference was not detected for (C).

Flower number significantly increased with plant size, *i.e.* stem volume, with a significant interaction between sex morph and plant size (Table 2, Fig. 2A). Fruit number also increased with plant size, and females set more fruits than did hermaphrodites (Fig. 2B). The total reproductive investment per plant, the sum of flower and fruit mass, significantly increased with plant size, with no significant difference between the sex morphs (Fig. 2C). Flower and fruit mass were calculated based on the mean dry mass of individual flowers (see Table 4) and fruits (72.2 ± 2.0 SE mg, *n* = 34; note that there was no significant difference in fruit mass between the sex morphs in our preliminary observation).

**Table 2:** Results of GLMs for evaluation of relationships between stem volume and reproduction. Best-fit models are indicated.

Variable	Coefficient	SE	*t*, *z* value*	*P* value
**Flower number**				
Intercept (Female)	1.54	0.20	7.78	**<0.001**
*ln* (Stem volume)	0.55	0.07	8.26	**<0.001**
Sex (Hermaphrodite)	−0.26	0.26	−1.02	0.31
*ln* (Stem volume) × Sex	0.21	0.09	2.44	**0.01**
**Fruit number**				
Intercept (Female)	−0.94	0.60	−1.56	0.12
*ln* (Stem volume)	0.61	0.20	3.10	**<0.01**
Sex (Hermaphrodite)	−1.52	0.33	−4.67	**<0.001**
**Flower + Fruit mass**				
Intercept	3.71	0.19	19.23	**<0.001**
*ln* (Stem volume)	0.71	0.07	10.87	**<0.001**

* *z* value for flower and fruit number, *t* value for sum of flower and fruit mass.

**Table 3:** Results of GLMs for evaluation of size-dependent resource allocation. Woody mass means dry weight of stem and roots; shoot mass means dry weight of stem and leaves; vegetative mass means dry weight of shoot and roots. Sample size is 21 for each sex morph. Best-fit models are indicated.

Variable	Coefficient	SE	*t* value	*P* value
**Flower mass**				
Intercept (Female)	3.33	0.22	14.94	**<0.001**
*ln* (Vegetative mass)	0.50	0.12	4.28	**<0.001**
Sex (Hermaphrodite)	0.48	0.16	3.04	**<0.01**
**Flower + Fruit mass**				
Intercept (Female)	3.59	0.21	17.13	**<0.001**
*ln* (Vegetative mass)	0.48	0.11	4.32	**<0.001**
Sex (Hermaphrodite)	0.28	0.15	1.90	0.07
**Leaf mass**				
Intercept	−1.82	0.10	−18.22	**<0.001**
*ln* (Woody mass)	0.65	0.06	10.36	**<0.001**
**Root mass**				
Intercept (Female)	0.86	0.13	6.61	**<0.001**
*ln* (Shoot mass)	0.57	0.13	4.50	**<0.001**
Sex (Hermaphrodite)	−0.18	0.17	−1.05	0.30
*ln* (Shoot mass) × Sex	0.38	0.20	1.96	0.06

**Table 4:** Comparisons of floral and vegetative traits between hermaphrodite (H) and female plants (F). mean ± SE is indicated with GLM results. Sample size is 21 for each sex morph.

	*F*	H	*t*, *z* value*	*P* value
**Floral traits**				
Flower mass (mg)	6.11 ± 0.27	8.96 ± 0.32	6.80	**<0.001**
Flower width (mm)	11.10 ± 0.20	15.76 ± 0.38	11.59	**<0.001**
Tube length (mm)	6.55 ± 0.13	8.47 ± 0.21	8.06	**<0.001**
Flower No.	11.4 ± 1.5	11.1 ± 1.6	–0.14	0.89
**Vegetative traits**				
Leaf mass (mg)	20.92 ± 1.47	20.60 ± 1.72	–0.14	0.89
Leaf size (cm^2^)	4.00 ± 0.27	3.57 ± 0.30	–1.05	0.30
Leaf No.	24.6 ± 2.8	23.6 ± 3.3	–0.25	0.80
Root mass (g)	3.81 ± 0.66	3.41 ± 0.55	–0.48	0.64

* *z* value for flower number and leaf number, *t* value for other traits.

### Effect of current reproduction on future reproductive effort

**Figure 3:**
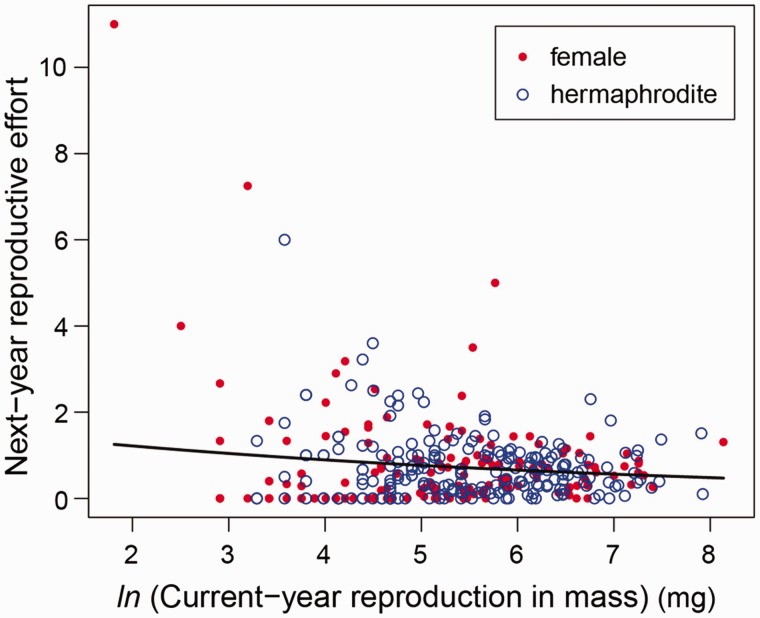
Relationships between the current-year investment in reproduction (sum of flower and fruit production in mass) and the next-year reproductive effort (relative flower production = flower number in the next year/flower number in the current year).

The current investment in reproduction (the sum of flower and fruit mass) hindered the relative flower production in the following year, indicating a trade-off between current and future reproduction (*z* = –3.64, *P* < 0.001; Fig. 3). Sex morph effect was excluded by AIC, indicating a lack of sexual difference in the cost of reproduction between hermaphrodites and females under natural conditions in this population.

### Fruit production under hand-pollination

Cross-pollinated female flowers set more fruits than cross-pollinated hermaphrodite flowers (60.4 ± 6.3 % and 21.1 ± 6.8 %, respectively), and these values were much higher than fruit-set rates under natural pollination (*z* = 14.76, *P* < 0.001, and *z* = 12.26, *P* < 0.001, respectively; Table 1). This indicates a severe pollen limitation under natural conditions for both females and hermaphrodites (PL = 0.86 and 0.93, respectively). Self-pollinated hermaphrodite flowers set significantly fewer fruits (7.1 ± 3.6 %) than cross-pollinated hermaphrodite flowers (21.1 ± 6.8 %, *z* = –2.89, *P* < 0.01), indicating minimal self-compatibility in hermaphrodites.

### Resource allocation and morphological traits

**Figure 4:**
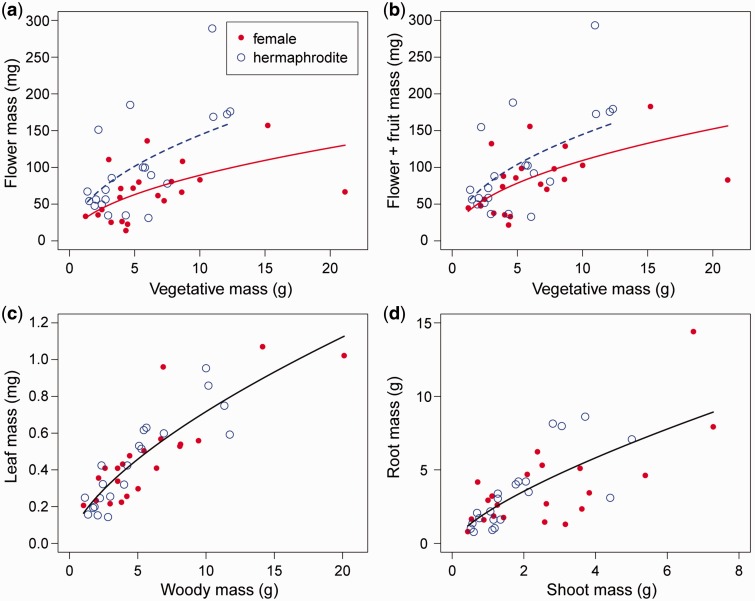
Relationships between plant size index and flower mass (A), flower + fruit mass (B), leaf mass (C), and relationships between shoot and root mass (D) for hermaphrodite (broken line) and female (solid line) plants. Woody mass means dry weight of stem and roots; shoot mass means dry weight of stem and leaves; vegetative mass means dry weight of shoot and roots. Sample size is 21 for each sex morph. Lines indicate the predicted relationships by GLMs. Sexual difference was significant for (A) and not significant for (B), (C), and (D).

Flower mass positively correlated with the total mass of vegetative organs, and hermaphrodites allocated more resources to flowers than females (Table 3, Fig. 4A). The total reproductive investment (the sum of flower and fruit mass) also increased with the total mass of vegetative organs, but did not significantly differ between the sex morphs (Fig. 4B). Sexual differences in resource allocation for the leaves and the root/shoot ratio were not detected in the GLMs (Fig. 4C and D).

Most floral traits showed significant sexual dimorphism, in which hermaphrodite flowers were larger in weight and size than female flowers, while the flower number was similar between hermaphrodite and female plants in our samples (Table 4). No vegetative traits varied significantly between the sex morphs.

### Photosynthetic capacity

**Figure 5:**
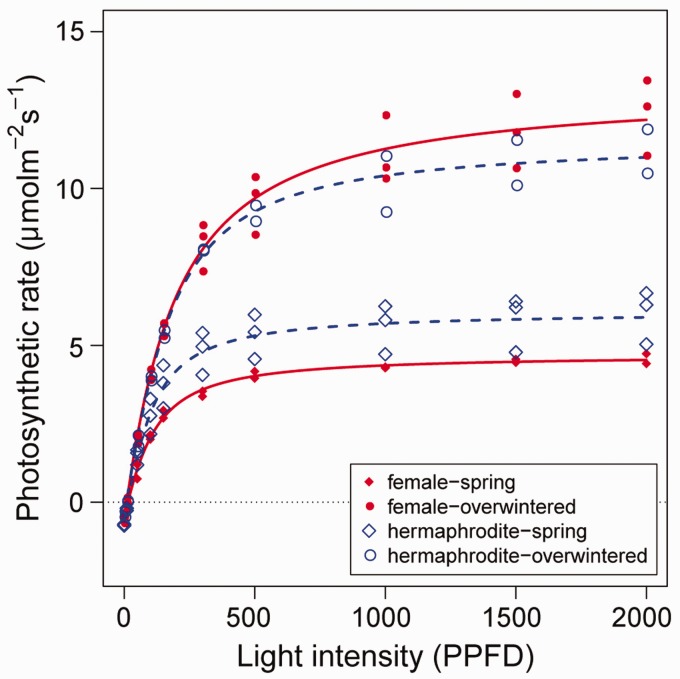
Light-photosynthetic curves in each leaf cohort of hermaphrodite (broken line) and female (solid line) plants. Sample size is two or three in each sex morph.

The light responses of photosynthetic capacity and the estimated light − photosynthetic curves were similar between the sex morphs in each leaf cohort (Fig. 5). Light-saturated photosynthetic rates ranged 5.2–6.8 and 12.0–13.8 µmol m^−^^2^ s^−^^1^, dark respiration rates ranged 0.52–0.72 and 0.62–0.46 µmol m^−^^2^ s^−^^1^, and the initial slopes of photosynthetic rates ranged 0.037–0.052 and 0.055–0.064 in spring and overwintered leaves, respectively. These results indicate the little difference in carbon fixation capacity between the sex morphs.

## Discussion

We observed significant sexual dimorphism in reproductive performance and size-dependent resource allocation in *D. jezoensis*. Hermaphrodites had larger flowers and produced more flowers as plant size increased, but they showed consistently less fruit production than females. Despite the apparent sexual difference in reproductive traits, hermaphrodites and females invested similar amount of resources in reproduction, and there were no differences in vegetative traits. We discuss the resource allocation strategy of each sex morph and the ecological factors affecting the maintenance of gynodioecy in this species.

We examined size-dependent flower production in hermaphrodites and fruit production in females. The reduced fruiting ability of hermaphrodites, independent of resource availability, indicates that hermaphrodites exhibit male-biased reproductive performance. Strong size-dependent flower production and large-sized flowers in hermaphrodites may contribute to improving success as a pollen donor (male fitness). The presentation of conspicuous flowers and/or a large display size with many flowers often attracts more pollinators and results in higher success as a pollen donor (Bell 1985; Vaughton and Ramsey 1998; Eckhart 1999; Williams *et al.* 2000; Van Etten and Chang 2014). The hand-pollination experiment revealed that the potential fruiting ability of hermaphrodites was one-third of females. In addition, the fruit-set rate of hermaphrodites by self-pollination (7 %) was much lower than that by cross-pollination (21 %), indicating very low selfing ability in this species. Kikuzawa (1989) reported that stigma clogging by self-pollen, *i.e.* the interference of outcross pollen receipt by autodeposition, strongly restricted the fruit-set success of hermaphrodites in this species. The very low fruit-set rate in hermaphrodites under natural pollination (< 2 %) may be caused by stigma clogging in addition to the potentially low fruiting ability in this population. Under such ecological constraints, hermaphrodites may gain fitness mainly through the male function as a pollen donor.

For females, size-dependent resource allocation to fruit production, rather than to flower production, contributes to fitness gain as a seed producer. In a natural population, however, the fruit-set rate of females (8 %) was much lower than the potential fruiting ability (60 %) because of severe pollen limitation. Pollen limitation is a ubiquitous phenomenon in animal pollinated plants in nature (Ashman *et al.* 2004; Knight *et al.* 2005). Low pollinator activity in early spring may cause severe pollen limitation for early bloomers (Alonso 2004). Since fruit-set success depends greatly on pollination success, resource investment in fruit production may vary in response to the extent of pollen limitation under natural conditions (Spigler and Ashman 2012; Van Etten and Chang 2014). Our study was conducted on a single population during one season, but severe pollen limitation has been reported in multiple populations of *D. jezoensis* (Kikuzawa 1989; Sinclair *et al.* 2016), suggesting that pollen limitation is common in this species. Factors other than pollen limitation, such as gender-specific herbivore attack, may affect the sexual difference in reproductive success of sexually dimorphic plants (Ashman 2002, 2006). However, evidence of gender-specific herbivore damage was limited in this species (personal observation), and the results of the pollination experiment strongly suggest the importance of pollen limitation in this population.

Irrespective of the significant sexual dimorphism in reproductive allocation, total resource investment in reproduction was similar between the sex morphs. This is an unexpected result from the view of different resource allocation strategies between hermaphrodites (as flower producers) and females (as fruit producers), because the individual mass of a fruit was 8–12 times larger than that of a flower. In addition, in several other gynodioecious species, a larger reproductive investment was detected in females when they produced more fruits than did hermaphrodites (Kohn 1989; Delph 1990; Vaughton and Ramsey 2011; Toivonen and Mutikainen 2012). Conversely, a similar resource investment in reproduction was reported between hermaphrodites and females of the gynodioecious *Sidalcea oregana* ssp. *spicata*, in which hermaphrodites produced as many fruits as females (Ashman 1994). Therefore, the relative fruit production of the individual sex morph is a key issue determining the sexual difference in the cost of reproduction. Under severe pollen limitation, restricted resource investment in fruit production of females may be balanced with a large investment of hermaphrodites in flower production. Although we detected significantly negative correlation between current-year investment in reproduction and next-year reproductive effort, *i.e.* cost of reproduction (Fig. 3), sexual difference was not detected. Similar resource investment in reproduction between the sex morphs may result in a similar cost of reproduction between hermaphrodites and females in this population. In populations with higher pollinator activity, however, resource investment in reproduction may be larger in females, resulting in more resource limitation for the next-year flower and/or fruit production in females. Therefore, a different scenario of sexual strategies is possible depending on pollination situation specific to populations (*e.g.*Toivonen and Mutikainen 2012). Further studies across multiple populations are necessary to make generalisations on the ecological significance of pollen limitation in this species.

In contrast to the clear sexual dimorphism in reproductive traits, we did not detect any difference in the vegetative and physiological traits (plant size, leaf size, and number, allocation to assimilative organ, aboveground vs. belowground allocation, and photosynthetic capacity) between the sex morphs. This result is in agreement with that of previous studies showing modest sexual differences in vegetative and physiological traits of gynodioecious species (Schultz 2003, 2009; Ashman 2005; Van Etten *et al.* 2008). Similar leaf traits, allocation to vegetative organs, and photosynthetic capacity between the sex morphs indicate that hermaphrodites and females may have similar assimilative, resource uptake, and storage capacities at the whole plant level. A lack of sexual dimorphism in vegetative traits is understandable in terms of source function of vegetative organs because total investment in reproduction was similar between hermaphrodites and females in this population. In our previous study conducted in another population, we reported that the maximum leaf size (but not the mean size per plant) was larger in hermaphrodites than in females (Sinclair *et al.* 2016). Genetic factors as well as ecological factors may be related to the sexual dimorphism in vegetative traits (Ashman 2005). If there is the genetic covariance between leaf size and flower size or number, for instance, hermaphrodites producing larger and more flowers may have larger leaves than that in females. Resource requirements for reproduction may also vary temporally between the sex morphs during the reproductive period (Delph 1990, 1999). To clarify the sexual difference in resource use dynamics, further studies are needed on the flowering, fruiting and leafing phenology of this species.

## Conclusions

The present study showed that hermaphrodites and females of *D. jezoensis* showed different size-dependent sex allocation. To our knowledge, this is the first study comparing the resource allocation pattern between the sex morphs at the whole plant level in a gynodioecious species. Male-biased reproductive allocation and low fertility in hermaphrodites support the conclusion from our previous study that gynodioecy in *D. jezoensis* may be close to a dioecious mating system (Sinclair *et al.* 2016). Despite the sexual difference in sex allocation, total reproductive investment did not differ between the sex morphs because of restricted female fruit production caused by pollen limitation. This resulted in no sexual differences in the cost of reproduction and other morphological and physiological traits. This study suggests that pollen limitation is important in maintaining the balanced resource investment in reproduction between the sex morphs under natural conditions.

## Sources of Funding 

This study is supported by JSPS KAKENHI grant Number 15H02641.

## Contributions by the Authors

All authors planned and led the project, conducted fieldwork, and contributed to data analyses and manuscript preparation.

## Conflict of Interest Statement

None declared.
